# Painter’s Colic (Colica Pictonum)

**Published:** 1878-05-20

**Authors:** O. C. Newton

**Affiliations:** Cincinnati


### PAINTER'S COLIC (COLICA PICTONUM.

BY O. C. NEWTON, M.D., OF CINCINNATI.

There are persons who are very liable to this disease; those who work in and with lead, occupants of lead manufactories; the business of painting generally.

A young man, twenty years of age, a painter by profession, had been in severe convulsions for two days, bowels constipated and enormously distended, and no movement accomplished, notwithstanding every effort had been used by previous physicians.

I was called at 2 p. m., found him in the above condition, in great agony.

Treatment: The first thing I did was to give him a full dose of morphia, one-third of grain hypodermically, with a view of relaxing his system, which had the desired effect, This I at once followed with castor oil internally, three tablespoonful, and one teaspoonful of turpentine, mixed, and taken in warm milk. Very soon after this, I gave him, through an anal gum elastic tube, passing it up the bowels as far as possible, a large injection of oil, four tablespoonsful, molasses, two tablespoonsful, and two teaspoonsful of salt in three pints of warm castile soap suds, holding the rectum to retain it; I also ordered a large warm hop poltice over the bowels. In less than an hour he had a copious discharge from the bowels.

I gave him the morphia in the way to relax the system, to allow the cathartic and the injection to do the work which it had failed to do with the other physicians. I followed this treatment by poltices and soothing aperients, and he soon recovered.

The point of superiority of practice in this case lay in the use of medicine by the hypodermic syringe, which did not nauseate, and which so relaxed the muscular contraction as to allow the balance of the treatment to do what it would not do with the muscular rigidity present.

Two pounds of chloroform had been given to this case, by inhalation, previous to my being called, but as soon as he came from under the influence of it, the spasms would return.

When I find persons who are working in paint, I instruct them to keep vessels filled with fresh water around them, especially house-painters. Such persons should drink freely of sweet milk—drank in the place of water. They should wear clothing free from the saturation of paint, contrary to their usual custom of using the same outside clothing, week in and week out. Bathing is highly necessary. If working in factories, they should have all the fresh air and ventilation possible.

There are some persons who cannot stand the least influence of paint, and such persons should not be permitted to work about it. Many persons will suffer with their kidneys, while others will suffer from a catarrhl affection, if they remain in a house that is being painted.

Every family in which there is a member who is subject to colic of any kind, should always possess themselves of and hold in readiness that valuable domestic appliance, "Davidson's rubber syringe," that they may have the benefit of an injection at once. It cannot be too promptly used. Very frequently, and in almost every case of colic, if the injection be early enough used, relief is the result.

